# The Quality of Alcohol Products in Vietnam and Its Implications for Public Health

**DOI:** 10.3390/ijerph6082090

**Published:** 2009-07-27

**Authors:** Dirk W. Lachenmeier, Pham Thi Hoang Anh, Svetlana Popova, Jürgen Rehm

**Affiliations:** 1 Chemisches und Veterinäruntersuchungsamt (CVUA) Karlsruhe, Weissenburger Strasse 3, 76187 Karlsruhe, Germany; 2 HealthBridge Vietnam, No. 15-16, 232 Lane, Ton Duc Thang Street, Dong Da District Hanoi, Vietnam; E-Mail: phanh@healthbridge.org.vn; 3 Centre for Addiction and Mental Health (CAMH), 33 Russell Street, Toronto, ON, M5S 2S1, Canada; E-Mails: lana_popova@camh.net (S.P.); jtrehm@aol.com (J.R.); 4 Dalla Lana School of Public Health, University of Toronto, 55 College Street, Toronto, ON, M5T 3M7, Canada; 5 Factor-Inwentash Faculty of Social Work, University of Toronto, 246 Bloor Street W, Toronto, ON, M5S 1A1, Canada; 6 Institute for Clinical Psychology and Psychotherapy, TU Dresden, Chemnitzer Strasse 46, 01187 Dresden, Germany

**Keywords:** alcoholic beverages, Vietnam, unrecorded alcohol, product quality, alcohol poisoning, traditional Chinese medicine

## Abstract

Four homemade (artisanally manufactured and unrecorded) and seven commercial (industrially manufactured and taxed) alcohol products from Vietnam were collected and chemically analyzed for toxicologically relevant substances. The majority of both types had alcohol contents between 30 and 40% vol. Two homemade samples contained significantly higher concentrations of 45 and 50% vol. In one of these homemade samples the labeled alcoholic strength was exceeded by nearly 20% vol. All other analyzed constituents of the samples (e.g., methanol, acetaldehyde, higher alcohols, esters, metals, anions) were found in concentrations that did not pose a threat to public health. A peculiarity was a homemade sample of alcohol with pickled snakes and scorpions that contained 77% vol of alcohol, allegedly used as traditional Chinese medicine. Based on this small sample, there is insufficient evidence to conclude that alcohol quality, beyond the effects of ethanol, has an influence on health in Vietnam. However, future research with larger samples is needed.

## 1. Introduction

Recently, the WHO has stressed the increasing importance of life-style related diseases for Vietnam [1]. Alcohol is an important risk factor for these disease categories [2]. Data from hospital statistics demonstrate a significant increase in alcohol-attributable diseases during the past decade, particularly in alcohol-related psychoses usually linked with dependence [3]. Other population research studies have demonstrated that alcohol plays an important role in causing chronic hepatitis, cirrhosis and hepatocellular carcinoma [4,5]. According to a report of the Minister of Transportation on a recent National Workshop on “Alcohol and road accidents” 10% of road accidents in Vietnam were caused by alcohol. Alcohol was detected in the blood of 62% of road accident victims [6]. Alcohol consumption has also been correlated with a higher risk of work-related injuries [7], as well as a higher prevalence for sexually transmitted diseases [8,9].

The most recent data on alcohol in Vietnam come from the 2004 Global Status Report on Alcohol, which estimated average per capita alcohol consumption for adults (defined as older than 15 years old) at about 2.4 liters of pure alcohol, of which 1 liter was considered to be unrecorded. While consumption still is markedly lower than global average consumption [10], it had been increasing in past years. Most importantly, however, the contribution of unrecorded alcohol is high and seems to be increasing [11], and thus many alcohol control measures such as taxation or labeling are impossible.

The most widely consumed alcoholic beverages were found to be spirits [12]. The prevalence of lifetime abstention was estimated at 69.5% (38.5% among men and 95.2% among women) [12]. Estimates of heavy drinking (defined as men who had more than 40 g of pure alcohol per day or women who had more than 20 g per day) placed the prevalence at 5.7% for men and 0.6% for women [12]. It was also estimated that among drinkers, the mean value of pure alcohol consumed per day was 16.4 g (total), 17.0 g in men and 8.6 g in women [12]. A recent epidemiological survey in rural Vietnam revealed that the prevalence of problematic drinking [measured by The Alcohol Use Disorder Identification Test (AUDIT)] was 25.5% among men and 0.7% among women [13]. The AUDIT is the most widely used measure to define problematic use worldwide, and usual norms were used [14].

Unrecorded alcohol products are defined as homemade alcohols (informally produced), illegally produced or smuggled alcohol products, as well as surrogate alcohol that is not officially intended for human consumption [15,16]. In Vietnam, the most common unrecorded alcohol is ‘rice wine’. This traditional form of alcohol has been produced communally by specialized villages from pre-colonial times, and is still considered a source of income for farmer families in rural areas [17,18]. The manufacture of this rice alcohol can be characterized as the conversion of rice (*Oryza sativa* L.) through physical, microbiological and biochemical operations, including steaming, inoculation with a starter, mashing and fermentation. The alcohol content of such ‘rice wines’ can reach alcohol volumes of up to 15% and 50% through distillation [18]. In Hanoi, the homemade alcohol usually is made from rice, in some regions it might also be made from corn or cassava.

There is a general lack of information concerning the composition of Asian alcoholic beverages in the scientific literature; with only limited overviews from China [19,20], Nepal [21], or Japan [22–24] available. Currently, there are no data available on health-relevant constituents and contaminants such as acetaldehyde, higher alcohols, metals, anions, or ethyl carbamate for either recorded or unrecorded alcohol products in Vietnam. The present study is the first pilot project to research alcohol quality in Vietnam, and its potential links to health outcomes. The study is part of our ongoing investigation of unrecorded alcohol from different countries, in which we already published results from Nigeria [25], Mexico [26], Lithuania and Hungary [16], Guatemala [27] and Poland [28].

## 2. Methods

### 2.1. Collection of Samples

**Figure 1.**
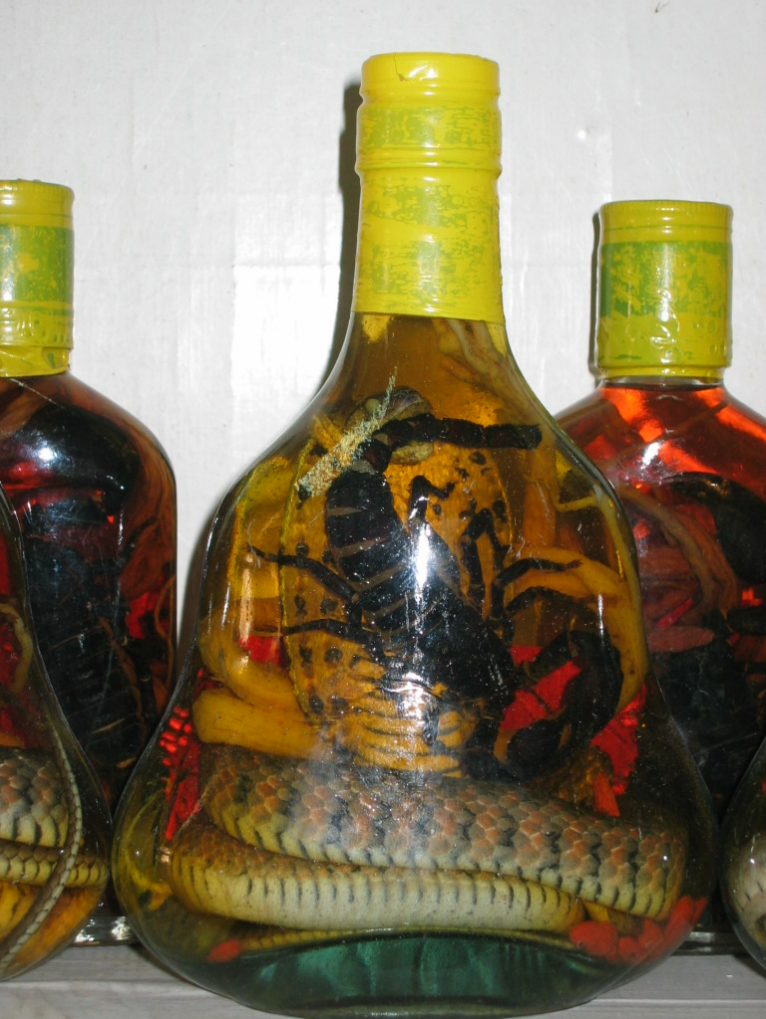
Homemade alcohol sample from Vietnam containing pickled snakes and a scorpion.

Eleven samples of alcohol products were collected by one of the authors (PTHA) from local markets in Vietnam and subsequent chemical analyses were conducted; all samples were locally produced, see Table 1 for details. Collection of alcohol products was based on a convenient sampling procedure aimed at obtaining products most popular with the population. Samples V03 and V05 are from the biggest, most popular manufacturers in the country, while V08 and V09 were homemade (traditionally produced) and are very popular in Vietnam. The designation “homemade” in this context does not mean that individuals prepared the alcohol for personal or family consumption. There are in fact some rural regions around Hanoi famous for producing specific homemade alcohols and providing them to the capital city via a system of commercial outlets, including local street markets, tea shops, or restaurants. Sample V11 was exceptional because it contained whole bodies of snake and scorpion soaked in alcohol (Figure 1); it was also homemade. Small, local companies produced the other samples.

### 2.2. Analytical Procedure

The parameters for chemical analysis were selected on a risk-oriented basis as described in previous studies [16,25,28]. Alcoholic strength was determined by Fourier Transform Infrared (FTIR) spectroscopy [29]. The analysis for volatile components was based on the European Community Reference Methods for the Analysis of Spirits using gas chromatography (GC) with a flame-ionization detector (FID) [30]. Additional details on the GC-FID procedure are published elsewhere [31]. Ethyl carbamate (urethane) was determined using GC with tandem mass spectrometry (GC-MS/MS) [32]. Anionic composition was analyzed using ion chromatography [33]. Conductivity was measured using the procedure outlined in Lachenmeier *et al.* [34]. Inorganic elements were analyzed using semi-quantitative inductively coupled plasma mass spectrometry (ICP-MS) after evaporation of the sample and re-constitution in ultrapure water. Furthermore, all samples were screened for unknown substances using GC with mass spectrometry (GC-MS).

### 2.3. Indication of Results

Alcoholic strength is indicated by ‘percent by volume’ (% vol). In accordance with the procedure described in the European Community Reference Methods for the Analysis of Spirits, volatile compounds contained in the samples are expressed by the unit ‘g/hL of pure alcohol’ or ‘g/hL of 100% vol alcohol’ (i.e., the concentrations are standardized in relation to alcoholic strength) [30]. This approach is superior to reporting in mg/L because the samples can be directly compared irrespective of their individual alcoholic strength. For clarity, we use the abbreviation ‘g/hL pa’. The results for the non-volatile components are presented as ‘mg/L’.

## 3. Results

The actual alcoholic strengths of the samples ranged from 29.5% vol to 76.7% vol. The highest alcoholic strength was found in the sample of alcohol that contained snakes and a scorpion (V11). Selected results from our chemical analyses are given in Table 2.

Methanol was detected in concentrations ranging from undetectable to 75 g/hL pa. Commercial samples labeled as vodka (e.g., samples V03 and V04) had the lowest methanol content, while higher levels were found in the brandy (V01) and rice alcohol (V10), and particularly the snake/scorpion-alcohol (V11) samples. In regards to levels of higher alcohols, content varied considerably between not detectable and 339 g/hL pa, a high variability was also found for ethyl acetate, ethyl lactate, and acetaldehyde.

The ICP/MS screening analysis for elemental composition showed that the most abundant elements, with concentrations in the mg/L range, were the alkali and alkaline earth metals, sodium, potassium, calcium, and magnesium. Other metals were found in traces below 1 mg/L.

The conductivities of the samples ranged from 1 μS/cm to 760 μS/cm. All samples were positive for chloride and sulphate, whereas nitrate and phosphate were detected only in some of the samples (Table 2).

Ethyl carbamate was only detected in the snake/scorpion-alcohol (V11) using GC-MS/MS (1.27 mg/L). During the multi-target screening analyses for unknown substances, conducted with GCMS, additional substances were also discovered solely in sample V11, where fatty acid ethyl esters such as ethyl palmitate, ethyl heptadecanoate, ethyl linoleate, ethyl oleate, ethyl stearate, ethyl arachidonate, were found in abundance, along with a number of sterols, with cholesterol being the most prominent.

## 4. Discussion

### 4.1. Alcoholic Strength

The majority of samples (72%) had an alcohol content between 30% vol and 40% vol, which is in accordance with the typical strength of international commercial spirits. The only abnormalities were the homemade samples V09 (45.0% vol) and V10 (49.7% vol), as well as the notable snake/scorpion-alcohol sample V11 with the highest strength of 76.7% vol. From a public health perspective V10 appears to be the most troubling of the samples as the labeling gave an alcoholic strength of 30% vol, whereas 49.7% vol were in fact contained in the sample - nearly 60% more alcohol than what was labeled, which could lead to severe intoxication and overdose [2].

The snake/scorpion sample V11 is discussed in greater detail below, however it should be noted in this case that the high alcoholic strength may be needed in order to preserve the pickled animals and subsequently increase the product’s shelf life. The alcoholic strength was not labeled on the sample; yet according to the local people we questioned, the common consumers are principally comprised of alcohol-dependent people of the lower socio-economic classes, who are well aware of the very high alcohol potency. Nevertheless, such a high strength alcoholic beverage may have more pronounced detrimental health effects explained entirely by the presence of ethanol [2].

### 4.2. Volatile Composition

Besides ethanol, our samples contained a number of volatile compounds, which are expected in products derived from alcoholic fermentation. Of these, methanol is the substance most often associated with the toxicity of surrogate and other alcohols [15]. As its presence was relatively low (i.e., lower than the European Union (EU) limit of 10 g/hL pa for vodka [35] in V01–V10), it would appear that in our samples, methanol content did not pose a threat to public health.

Acetaldehyde is a substance considered by the International Agency for Research on Cancer as ‘possibly carcinogenic to humans’ (IARC Group 2B) [38]. It is also regarded as an undesirable substance in spirits because of its unpleasant flavor. The acetaldehyde level in some of the samples was higher than the EU limit of 0.5 g/hL pa for vodka, but typical for spirits distilled under retention of the organoleptical properties of the raw materials (e.g., brandy, whisky, rum) [39]. The highest concentration of 19 g/hL pa, found in the homemade sample V10, can be considered typical for an artisanally produced spirit, as average acetaldehyde residues between 12 g/hL pa and 18 g/hL pa have been found, for example, in artisanal German fruit spirits [40].

Alcohol containing more than two carbon atoms is commonly called ‘higher’ or ‘fusel’ alcohol. Most higher alcohols are created as a byproduct of yeast fermentation and are an important flavor compound. In the EU, some spirits must have minimum contents of higher alcohols, whereas neutral alcohols such as vodka should be almost entirely free of higher alcohols (max. 0.5 g/hL pa) as well as of esters (max. 1.3 g/hL pa) [35]. In this sample group, two of the commercial vodkas (V04, V05) and the two homemade rice alcohols (V09, V10) were above the EU requirement for higher alcohols; the two homemade alcohols also above the requirement for esters. Conversely, the brandy (V01) and rum (V07) samples, were below the minimum EU requirements of 140 or 225 g/hL pa of higher alcohols, for the two groups of spirits respectively. In terms of higher alcohols, sample V10 was the most peculiar, as it had an extremely high content of higher alcohols. The high concentration of esters and ethyl lactate in this sample indicates probable hygienic deficiencies during fermentation (e.g., spoilage with acetic acid or lactic acid bacteria). The other homemade samples showed a surprisingly clean alcohol quality. In conclusion, none of the samples pose a public health risk due to the contents of higher alcohols [41].

### 4.3. Non-Volatile Compounds and Water Quality

Because elements and ions are generally non-volatile, most of the inorganic content found in the spirits is derived from the dilution water used to adjust the distillate to drinking strength. Inorganic contamination may also occur during the use of the distillation equipment. Other than the snake sample discussed below, all the alcoholic beverages conformed to the EU drinking water requirements [37]. The conductivities of the samples and the contents of ions were relatively low, which suggests an overall sufficient water quality or treatment process. No toxic anions such as nitrates, or toxic metals including lead, cadmium, copper, antimony, or arsenic, were found in health relevant concentrations.

### 4.4. Alcohol with Snakes and Scorpions – A Traditional Chinese Medicine?

The sample that was significant in nearly all analyzed parameters was sample V11, the alcohol containing pickled snakes (cobras) and a scorpion. Typically, the production of such products is comprised of the purchase of a live snake, usually from a street vender/shop or by pre-order, the snake is killed and then placed in a desired glass pot, which depends in volume, shape and style on the size of the snake. This process is carried out by both individuals in their homes, as well as by vendors in local markets in the presence of their customers, so usually there is no bottle label provided.

The use of snakes (e.g., cobras or other snakes) as human food is traditional in Asia [42]. The pickling of snakes in vinegar or in alcohol has been described as a common practice in China, mostly in and around Guangzhou (Canton) and in Taiwan; here the snake meat is used for dishes, while the liquid is seemingly only used for preservation purposes [43]. In the case of our sample however, the snake is not used for eating, but is used, along with the scorpion (including venomous ones), in order to provide an essence for purported medicinal benefit. This practice has its roots in Traditional Chinese Medicine (TCM), popular in Vietnam, where such an alcoholic beverage is used as a treatment for rheumatism and arthritis. In this system, it is believed that the more toxic the snake, the more beneficial; an example of this is “Dragon and Phoenix Wine”, a popular beverage in China, which is made using a venomous snake and a pheasant soaked in a distilled high-proof spirit [43].

There is only sparse information in the literature and there are no systematic studies, regarding whether the snake or scorpion venoms consumed orally as TCM (or during food use) pose a threat to public health. It is unlikely that the venom glands have been separated before infusion in the alcohol, yet the venom might be denatured by the high-proof alcohol. Snake venom has also been described as being much less toxic when administered orally rather than intravenously because it may be broken down by the digestive enzymes of the gastrointestinal tract, as well as hydrolyzed by the acidic conditions [44,45]. Recent research has shown that the use of animal products as TCM may be justified, but little research has been done to prove the claimed clinical efficacy [46].

In our screening analysis of the sample, we only found constituents of the animal fat (e.g., fatty acid esters or cholesterol). However, as we did not know the exact species of the snake or scorpion, we could not search specifically for the venom. The complex proteins in snake venoms would most likely not have been identifiable with our analytical methodologies anyway. Therefore, the slight possibility for a health risk of the product remains, even if intoxications of humans from snakes and scorpions in the food chain, to our knowledge, have never been reported. Besides the animal constituents, the sample contained a comparably high concentration of methanol, which, however, is still much below toxic concentrations [47]. Another exceptional characteristic of the snake/scorpion sample was the high conductivity and chloride level, which may be explained by the diffusion of chloride and other ions from the animals into the liquid or insufficient water quality. However, no anions or metals posing health risk were found in the snake sample (e.g., nitrate, lead, antimony, arsenic were below the EU maximum levels [37]).

An important finding is that the snake/scorpion alcohol also contained ethyl carbamate (urethane), a ‘probably carcinogenic’ substance (IARC Group 2A) [48], in relatively high concentrations above the Canadian limit [36]. Ethyl carbamate may contribute to carcinogenicity of alcoholic beverages [49] due to the synergistic effects between ethanol, ethyl carbamate and other possible carcinogenic contaminants of foods that are co-ingested with alcoholic beverages [50]. This is a surprising finding as ethyl carbamate is generally found in such high concentrations only in stone-fruit spirits [51]. The possibility cannot be excluded that some constituent of the snake or scorpion (e.g., nitrogen compounds or amino acids) may react with ethanol to form ethyl carbamate. The formation mechanism in this special kind of alcoholic beverages is far from clear, and requires further investigation.

## 5. Conclusions

Our research provides the first insight into the quality of alcohol in the markets and street shops in Vietnam. Overall, the quality of the homemade and industrially produced samples was relatively high. Specifically, except for ethanol, no compounds that may lead to acute toxic effects were detected. Although some problems with product quality, production hygiene and labeling were found, this pilot study cannot state that alcoholic beverages, both homemade and industrial, produced in Vietnam are associated with health risks beyond those of alcoholic beverages in the rest of the world.

There are several methodological limitations to our study. First of all, only a limited number of samples were analyzed. Secondly, because of the convenience sampling strategy, our samples only provide a cross-section of the situation in the capital city of Vietnam, Hanoi, while unrecorded home production may be particularly prevalent in rural areas. Given the small sample size, it might have excluded problematic alcohol products that were widely consumed.

We consider our study a pilot project that serves as a foundation for future research on the relationship between alcohol quality and health outcomes, in Vietnam and Asia, as there is a great disparity between the plethora of issues to be investigated and the literature that exists.

Future research should focus on the collection of large, representative alcohol samples, and include both rural and urban regions. Given the extent of alcohol-attributable disease burden in Vietnam and Asia, it is disappointing that so little research has been conducted on the health consequences related to alcohol and its quality; additionally, more research on sources and patterns of consumption is needed. Particular focus should be placed on unrecorded sources (e.g., homemade alcohol), which showed some quality problems in our study, especially the standardization of alcoholic strength. Finally, case control analyses for the most prevalent chronic and acute alcohol-related disease groups [2], including chemical analyses of alcohol products, should be conducted.

## Figures and Tables

**Table 1.** Alcohol samples collected in Vietnam (Hanoi).

**Sample**	V01	V02	V03	V04	V05	V06	V07	V08	V09	V10	V11
**Description** (Name of Beverage)	Brandy (Napoleon XO)	Whisky (Wall Street)	Vodka (Sai Gon)	Vodka (Ha Thanh)	Vodka (Hanoi)	Shochu (Yama)	Rum (Chauvet Blanc)	Rice alcohol (Sticky)	Rice alcohol	Traditional Rice alcohol (Ruou bau da)	Alcohol with snakes and scorpion
**Type of alcohol**	Commercial (Int. Alcohol Join venture Company (ISC))	Commercial (Allied Commerce Vietnam)	Commercial (Saigon Beer and alcohol and beverage Company)	Commercial (Ha Thanh Beer, Alcohol and Beverage Company)	Commercial (Hanoi Beer Company)	Commercial (Five Stars Exp-Import Company)	Commercial (Int. Alcohol Join venture Company (ISC))	Homemade	Homemade	Homemade	Homemade
**Labelled alcoholic strength [% vol]**	39	39	29.5	30	29.5	29	39	29.5	45	30	no label

**Table 2.** Composition of Vietnamese alcohol products in comparison to international standards.

**Component**	**V01**	**V02**	**V03**	**V04**	**V05**	**V06**	**V07**	**V08**	**V09**	**V10**	**V11**	**International Standards**

**Ethanol [% vol]**	38.2	39.3	30.4	29.5	30.7	30.2	39.2	30.8	45.0	49.7	76.7	–a
**Methanol [g/hL pa]**	4.9	1.2	nd	nd	1.1	3.3	1.4	1.6	nd	4.6	75	30 / 1,000 b
**Acetaldehyde [g/hL pa]**	9.4	6.4	nd	0.6	nd	14	0.9	1.2	nd	19	13	0.5 / no limit b
**1-Propanol [g/hL pa]**	6.1	7.2	nd	7.8	0.8	20	30	1.1	nd	43	2.1	–
**1-Butanol [g/hL pa]**	nd	nd	nd	nd	nd	0.5	nd	nd	nd	0.7	nd	–
**2-Butanol [g/hL pa]**	nd	nd	nd	nd	nd	nd	0.6	nd	nd	nd	nd	–
**Isobutanol [g/hL pa]**	8.9	12	nd	3.2	0.5	39.4	3.2	nd	nd	120	nd	–
**Amyl alcohols [g/hL pa]**	15	38	nd	0.6	nd	75	11	nd	nd	164	0.5	–
**1-Hexanol [g/hL pa]**	nd	nd	nd	nd	nd	nd	nd	nd	nd	0.5	nd	–
**2-Phenyl ethanol [g/hL pa]**	nd	nd	nd	nd	nd	nd	nd	nd	nd	11	nd	–
**Ethyl acetate [g/hL pa]**	3.8	4.6	nd	nd	nd	8.8	0.7	nd	nd	46	12	1.3 / no limit b
**Ethyl lactate [g/hL pa]**	1.3	0.8	nd	nd	nd	nd	nd	nd	nd	74	2.6	–
**Ethyl caprylate [g/hL pa]**	nd	0.5	nd	nd	nd	nd	nd	nd	nd	0.5	nd	–
**Sum of higher alcohols [g/hL pa]**	30	57	nd	12	1.3	135	45	1.1	nd	339	2.6	0.5 / no limit (minimum 200) b
**Ethyl carbamate [mg/L]**	nd	nd	nd	nd	nd	nd	nd	nd	nd	nd	1.27	0.4 c
**Conductivity [μS/cm]**	36	32	16	64	1	–	9	79	0	24	760	2,500 d
**Chloride [mg/L]**	2.1	2.1	3.2	2.2	0.5	–	0.7	0.5	0.7	1.0	296	250 d
**Nitrate [mg/L]**	nd	0.9	0.7	0.6	1.1	–	1.0	0.7	1.0	1.0	nd	50 d
**Phosphate [mg/L]**	nd	nd	nd	nd	nd	–	nd	nd	nd	nd	22.7	–
**Sulfate [mg/L**	4.6	3.8	3.0	2.5	1.2	–	1.4	0.8	3.0	1.8	37.5	250 d

^a^ No international standard available;

^b^ EU spirit standards (maximum level for neutral alcohol/for fruit spirits) [35];

^c^ Canadian standard for ethyl carbamate in fruit brandies and liqueurs [36];

^d^ EU drinking water quality standards [37]; nd: not detected (detection limits: volatiles 0.5 g/hL pa, chloride 2 mg/L, nitrate 5 mg/L, phosphate 10 mg/L, sulfate 5 mg/L, ethyl carbamate 0.01 mg/L). Negative in all samples: benzyl alcohol, methyl acetate, benzyl acetate, ethyl benzoate, benzaldehyde.
